# Tolvaptan-induced Liver Injury: Who is at Risk? A Case Report and Literature Review

**DOI:** 10.7759/cureus.4842

**Published:** 2019-06-05

**Authors:** Muhammad Y Khan, Muhammad Shabbir Rawala, Maryam Siddiqui, Waqas Abid, Aysha Aslam

**Affiliations:** 1 Internal Medicine, Unitypoint Health, Rock Island, USA; 2 Internal Medicine, Charleston Area Medical Center, Charleston, USA; 3 Interventional Radiology, Christiana Hospital, Newark, USA; 4 Internal Medicine, Louis A. Weiss Memorial Hospital, Chicago, USA

**Keywords:** hyponatremia, tolvaptan, tolvaptan, siadh, drug-induced hepatotoxicity

## Abstract

Hyponatremia is a common clinical condition encountered in the hospital setting. Syndrome of inappropriate antidiuretic hormone (SIADH) is an important and one of the most common causes of hyponatremia. SIADH accounts for approximately one-third of all cases of hyponatremia. Tolvaptan is a vasopressin receptor antagonist used to treat SIADH. Hepatoxicity is a rare yet dangerous side effect from Tolvaptan use. We present a case of cholestatic liver injury in an elderly female who presented with hyponatremia. She received two doses of tolvaptan 15mg and developed worsening in her total bilirubin (T Bili) and alkaline phosphatase (Alk Phos) levels. Tolvaptan is known to cause elevated transaminase levels and the mechanism of action is thought to be idiosyncratic. Fortunately, the patient responded with an improvement in T Bili and Alk Phos levels after stopping tolvaptan. This case highlights the cautious use of tolvaptan in elderly patients with SIADH as even small doses can potentiate hepatotoxicity.

## Introduction

Hyponatremia (defined as serum sodium <135 mmol/L) is a common clinical condition seen in the hospital setting. It is categorized as mild, moderate, and severe. The incidence of hyponatremia in hospitalized patients is reported to be approximately 15% to 30% [1-2]. Hyponatremia is considered an independent risk factor for mortality in the elderly population as it is associated with increased fall risk and prolonged hospital stay. Syndrome of inappropriate antidiuretic hormone (SIADH) is an important cause of hyponatremia in hospitalized patients and can be an initial presenting sign of an underlying malignancy [3]. Incidence of hyponatremia increases with age and is commonly seen in elderly and hospitalized patients. SIADH accounts for about one-third of all cases of hyponatremia [4-5]. Tolvaptan is a commonly prescribed drug used for the treatment of SIADH-induced hyponatremia. It is a vasopressin antagonist that acts on V2 vasopressin receptors. A rare but important side effect of tolvaptan is hepatotoxicity. Liver injury pattern characterized by tolvaptan is categorized as an idiosyncratic drug reaction and is characterized by the elevation in liver enzymes and biliary cholestasis. There are no clear guidelines to monitor liver function in patients who are treated with tolvaptan.

We present an interesting case of acute liver injury with cholestasis in a female patient after receiving two doses of tolvaptan for severe hyponatremia after a motor vehicle accident (MVA). The patients developed progressive worsening in liver enzymes with markedly elevated alkaline phosphatase and bilirubin levels after receiving two doses of 15 mg of tolvaptan. Liver function tests (LFTs) showed improvement in the alkaline phosphatase (Alk Phos) and bilirubin (T Bili) levels after stopping tolvaptan.

## Case presentation

An 86-year-old Caucasian female with past medical history significant for hypertension, chronic kidney disease (CKD) stage III, history of coronary artery disease (CAD), osteopenia, history of bladder cancer taking calcitriol, and history of squamous cell carcinoma on the right chin bone presented to the emergency department (ED) after being involved in a MVA. Initial laboratory workup showed severe hyponatremia with the sodium level of 118 mEq/dl. The patient did not have any neurological symptoms on presentation. Initial computed tomography (CT) scan of the head was unremarkable for acute intracranial abnormality. The patient denied taking any anti-psychotic or anti-seizure medications. She was recently prescribed a course of sulfamethoxazole-trimethoprim that she took for three weeks for a lower-extremity wound infection. She stopped the antibiotic four weeks before her current hospitalization. She was admitted for the management of severe hyponatremia and received fluid resuscitation with 0.9% NaCl in the ED.

Nephrology service was consulted for severe hyponatremia. Urine studies showed urine osmolality 267 mOsm/kg, urine sodium 30 mmol/L, and urine creatinine 62.2 mg/dl. Her lab work was consistent with a diagnosis of SIADH. The patient was placed on fluid restriction. Since the patient did not show much improvement in the sodium levels with fluid restriction and diuretics, she was started on tolvaptan by nephrology service. The patient received tolvaptan 15 mg daily for two doses as per nephrology recommendations.

LFTs were checked the following day after receiving the first dose of tolvaptan showed alanine aminotransferase (ALT) levels of 193 U/L, aspartate aminotransferase (AST) 132 U/L, Alk Phos 424 U/L, T Bili 2.8 mg/dl with direct T Bili 2.3 mg/dl. The patient had normal LFTs before admission two months ago. She received two doses of tolvaptan during her current hospital stay and there was progressive worsening in her LFTs with T Bili of 8.2 mg/dl, Alk Phos 815 U/L, ALT 255 U/L, and AST 155 U/L. Tolvaptan was discontinued, and the patient underwent further workup to evaluate for causes of hyperbilirubinemia and elevated liver enzymes. Ultrasound (US) of the abdomen was unremarkable for gallstones or common bile duct (CBD) dilatation. Kidneys were normal and did not show adult polycystic kidneys (ADPKD). The gamma-glutamyl transferase (GGT) level was 691 U/L. She had normal coagulation studies. Due to the progressive worsening in her LFTs, the gastroenterology service was consulted on day 7 of admission. She underwent workup for chronic liver disease (CLD) and autoimmune hepatitis including negative anti-nuclear (ANA) antibodies (Ab), anti-smith Ab, and anti-mitochondrial Ab. Hepatitis serologies were negative for hepatitis B surface antigen [i](Hep B sAg), hepatitis B core Ab (Hep B core IgM), hepatitis C Ab, and hepatitis A IgM. Magnetic resonance imaging of the abdomen (MRCP) that was negative for choledocholithiasis did not show any evidence of liver cirrhosis. On day 9, LFTs eventually started to show improvement and a downward trend. The patient was cleared for discharge by GI service and advised to follow up as outpatient for close monitoring of her LFTs. On the day of discharge, the T Bili levels decreased to 5.5 mg/dl, ALT 164 U/L, AST 102 U/L, and Alk Phos 815 U/L. She remained asymptomatic.

## Discussion

Tolvaptan is a vasopressin receptor antagonist, which works by acting on V2 receptors of the collecting tubules. It acts by causing selective water diuresis without affecting electrolyte excretion and is commonly used for the treatment of hyponatremia (sodium level <130 meq/l) [6]. Tolvaptan and several other agents have been approved by FDA for use in severe hyponatremia in May 2009 with recommendations for use including not using it more than 30 days and should not be given to the patients with underlying liver disease [7].

Liver injury caused by tolvaptan is well established in patients with ADPKD as demonstrated by RCTs in the past. Torres et al. demonstrated that out of 961 patients, 1.5% discontinued the drug due to liver dysfunction, 4.5% have a clinically significant increase in the levels of aminotransferases, and 0.9% had an increase in the bilirubin levels. This study demonstrated the potential benefit of tolvaptan use in slowing the progression of renal disease in patients with ADPKD. This study and its extension of an open-label study indicated three cases of severe liver injury attributed to this drug, leading to liver failure [8]. There has been a significant concern for the use of tolvaptan in patients with ADPKD as demonstrated by Tolvaptan Efficacy and Safety in Management of Autosomal Dominant Polycystic Kidney Disease and Its Outcomes (TEMPO 2:3 trial). Approximately 10.9% of patients in the tolvaptan group had adverse hepatic events compared to 5.3% in the placebo group. The most common manifestation was ALT elevation three times the upper limit of normal. All cases showed resolution in LFTs after stopping tolvaptan [9].

The present case is interesting as it points out towards idiosyncratic liver injury caused after receiving only two doses of tolvaptan 15 mg for SIADH. Similarly, the timing and pattern of hepatic injury are also unusual from the previously reported data on tolvaptan-induced liver injury. Tolvaptan usually presents with elevation in AST and ALT levels and cholestasis picture is not commonly associated with its use. The workup for abnormal LFTs did not show evidence of acute or chronic hepatitis, autoimmune, biliary obstruction, or cirrhosis in our patient. The clinical course showed the progressive worsening in LFTs with a predominant cholestasis picture with gradual improvement after stopping tolvaptan. Another important factor to consider is that the patient finished trimethoprim-sulfamethoxazole treatment four weeks ago before her presentation. Trimethoprim-sulfamethoxazole is associated with cholestatic or mixed type with both cholestasis and parenchymal injury. The onset of symptoms can happen from a few days to a month after therapy commencement. Literature has shown resolution in symptoms and LFTs levels generally within six months [10-12].

Tolvaptan-induced liver injury is characterized as an idiosyncratic drug reaction. The underlying cause could be genetic and non-genetic susceptibility factors. The major risk factor identified is ADPKD. Usually, the liver injury caused by tolvaptan occurs after 3-18 months, but from our case mentioned above, this might not be necessary [13]. The exact pathogenesis of tolvaptan-induced liver injury is not known, however previous literature, animal studies, and RCTs have given some insight about the proposed mechanism. An important animal study conducted by Mosedale et al demonstrated genetic susceptibility as one of the most important mechanisms for idiosyncrasy [14]. Genetic mapping and transcriptomic analysis were performed in the liver of the mice population who developed elevations in liver enzymes and significant liver injury. Variation in the functioning of important enzymes identified in the pathway involved epoxide hydrolase, interferon regulatory factor 3 and mitochondrial fission factor was based on genetic susceptibility. There was also significant variation in oxidative stress and immune response pathways and bile acid homeostasis as indicated by gene analysis. Moreover, it was determined that the oxidative stress pathway and innate immune response pathways were induced in all the strains exposed to the drug; however, bile homeostasis was significantly impaired in those with drug-induced liver injury (DILI). Hence, loci on chromosome 14 were identified to cause significant stress response and alterations in the bile acid homeostasis. This study also gave an insight into the dosage response to tolvaptan in mice with DILI. Plasma drug concentrations were minimally related to the degree of liver response [15-16]. This study provides an important insight about DILI caused by tolvaptan and may open avenues for new biomarkers to be identified in the coming future which can be used as an early surrogate to identify ppl at risk for this idiosyncratic reaction in individuals with exaggerated stress response based on genetic susceptibility. As indicated by previous studies and FDA warning, tolvaptan induced liver injury can potentiate to progressive liver failure, transplant or even death of the patient. Besides ADPKD, the exact cause which might put the patient at risk for this kind of complication is unknown. More studies are needed to look into exact pathophysiology of liver damage in this case. It could be related to individual factors like genetic susceptibility in some patients as to how they metabolize the drug, which in some individuals can produce more oxidative damage compared to others.

Given the hepatic adverse effects of tolvaptan in a large multicenter TEMPO trial in ADPKD patients, close monitoring of these patients is recommended by the FDA. However, this drug has the potential to cause liver injury in patients without underlying liver disease or ADPKD, as seen in our case report. Guidelines for monitoring of liver function tests need to be reconsidered based on future studies including larger patient population. Currently, these patients should be monitored at one- to three-month interval to see the resolution or progression of liver dysfunction in these cases to indicate if liver dysfunction was directly related to drug use and not progression of already existing pathology.

## Conclusions

Tolvaptan is commonly used to treat patients with SIADH. Hepatotoxicity is a rare yet serious side effect associated with tolvaptan use. Reviewing the medication history of patients to exclude potential hepatotxic drugs is very important. One should obtain baseline LFTs before starting on tolvaptan therapy. Special caution should be exercised in elderly patients as even smaller doses of tolvaptan can lead to progressive worsening of liver enzymes and possible liver failure. Recent use of potential hepatotoxic drug must also be kept in consideration as potential drug reactions may result in unpredictable patterns of liver injury.
